# Paradoxical Inadequate Parathyroid Hormone Secretion Secondary to Severe Hypomagnesemia: A Review of the Literature

**DOI:** 10.1016/j.xkme.2025.101046

**Published:** 2025-06-14

**Authors:** Annemarie Albert, Ulrich Paul Hinkel, Theresa Bohlender, Philipp Stieger, Rüdiger C. Braun-Dullaeus, Christian Albert

**Affiliations:** 1 Department of Nephrology, Central Clinic Bad Berka, Bad Berka, Germany; 2 University Clinic for Cardiology and Angiology, Otto-von-Guericke University Magdeburg, Magdeburg, Germany

**Keywords:** Hypoparathyroidism, hypomagnesemia, hypocalcemia, calcium-sensing receptor, parathyroid gland, electrolyte, calcium, magnesium homeostasis, hypoparathyroidism paradox

## Abstract

Hypocalcemia is a common manifestation frequently encountered secondary to hypomagnesemia. Both calcium and magnesium are essential for maintaining normal cellular physiology, and magnesium plays a pivotal role in modulating neuronal excitation, intracardiac conduction, and myocardial contraction by regulating several ion transporters. Associated disorders may include cardiac arrhythmia, heart failure due to insufficient contractility, and neuromuscular and central nervous system conditions with seizures. One of the most important factors underlying hypocalcemia in hypomagnesemia conditions is the impaired secretion of parathyroid hormone (PTH), referred to as paradoxical hypoparathyroidism. Because there is a positive functional correlation and association between serum magnesium and calcium concentrations, clinical hypocalcemia in cases of magnesium deficiency cannot be sufficiently corrected by supplementation with calcium, vitamin D, or both. In contrast to the clinical relevance of a rapid, consequent and effective detection, differential diagnosis, and subsequent initiation of an appropriate therapy, this phenomenon and underlying pathophysiology are not well understood. In this review we summarize on calcium and magnesium homeostasis through modulation of PTH and vitamin D and elaborate on the mechanism underlying the rare condition of paradoxical inadequate PTH secretion. Based on the relevant literature, our review includes interdisciplinary diagnostic and therapeutic recommendations.

Hypomagnesemia is extremely common, affecting up to 12% of all hospitalized patients and up to 60% of patients in the intensive care unit.^1^ Hypomagnesemia is associated with an increased risk of certain chronic conditions, such as metabolic syndrome, type 2 diabetes mellitus, and cardiovascular diseases.^2^ Most causes of hypomagnesemia are due to gastrointestinal or renal loss.^3^ But also commonly used drugs such as proton pump inhibitors decrease intestinal magnesium absorption, whereas chronic use of thiazide diuretics are known to increase renal magnesium wasting independent of the estimated glomerular filtration rate (eGFR).^1^

Both blood ionic magnesium (Mg^2+^) and ionic calcium (Ca^2+^) regulate the secretion of parathyroid hormone (PTH) from the parathyroid gland in form of a feedback mechanism; however, the potency of the former is suggested to be less than that of Ca^2+^.^4^ Several pathologies can be responsible for the dysregulation of PTH–either being too high or too low–referred to as hyperparathyroidism or hypoparathyroidism, respectively. Even mild degrees of magnesium depletion may already cause a significant decrease in serum calcium levels.^5^

In conditions with hypocalcemia associated with hypomagnesemia, inadequate low or inappropriate normal PTH secretion has been observed, referred to as paradox inhibition of PTH secretion associated to hypomagnesemia. However, little is known about the underlying pathophysiology. Regarding the complexity of the pathophysiology, specifically disciplines not caring for patients with electrolyte disorders on a regular basis may benefit from a care-oriented overview. Therefore, this review aims to identify and summarize the available evidence on impaired PTH secretion despite hypocalcemia secondary to magnesium deficiency. Interacting metabolic processes are outlined and conclusions for diagnostics, therapy, and patient management are drawn.

## Methods

The initial approach of a systematic review of the literature was discarded because a systematic search performed using the keywords listed below revealed that there was not enough primary literature available to systematically summarize and elaborate on the pathophysiology of this phenomenon.

With the primary focus on deepening the understanding of the clinical condition, we therefore opted to conduct a non-systematic, structured narrative literature review using electronic searches of the Medline (via PubMed interface), Google Scholar, and Scopus databases to identify relevant publications.^6^ The search targeted reviews, case report and primary studies investigating the relationship between magnesium, calcium and PTH using keywords such as “magnesium” or “Mg2+,” “calcium” or “Ca2+,” “hypocalcemia,” and “hypomagnesemia,” “parathyroid hormone,” “PTH” or “parathormone” along with “paradox,” “inhibition,” “functional” or “inadequate” including all studies ever published through June 2024. In addition, focused searches and review of reference lists from identified relevant publications were subsequently performed to further explore the possible underlying mechanisms and biologic plausibility beyond that reported in reviews or case study publications. Finally, this review includes diagnostic and therapeutic recommendations based on findings from the relevant literature and our own patient experience. Based on these findings, figures were created de novo to illustrate the underlying pathology. Causes of hypomagnesemia were extracted from the literature and summarized in Table 1.

**Table 1:** Causes of Hypomagnesemia

Magnesium redistribution from the extracellular to the intracellular compartment
Refeeding syndrome/insulin
Hungry bone syndrome
Correction of metabolic acidosis
Treatment of diabetic ketoacidosis with insulin
Catecholamine excess
Extensive blood transfusions
Acute pancreatitis
Alcohol withdrawal syndrome
Intravenous glucose /intravenous hyperalimentation
Osteoblastic metastases
Lactation
Gastrointestinal causes
Reduced intake
Magnesium free intravenous fluids
Dietary deficiency low oxalate diet cellulose phosphate
Gastrointestinal fistulas
Inflammatory bowel disease
Vomiting
Reduced absorption
Malabsorption syndrome (sprue, steatorrhoe)
Acute diarrhea
Chronic diarrhea (Crohn disease, chronic enterocolitis ulcerosa)
Intestinal resection (short bowel syndromes, but also gastric bypass surgery)
Primary infantile hypomagnesemia
Nasogastric suction
Renal loss
Reduced sodium reabsorption
Saline infusion
Thiazide diuretics, osmotic diuresis, less so loop diuretics
Diabetes mellitus
Excessive vitamin D intake
Ketoacidosis
Hypercalcemia/hypophosphatemia
Potassium depletion
Phosphorous depletion
Renal diseases and acquired tubular dysfunction
Tubulointerstitial renal diseases
Secondary due to hyperfiltration syndromes
Post obstructive nephropathy
Diuretic phase of acute kidney injury
Post kidney transplantation
Renal tubular acidosis
Dialysis
Inherited disorders
Gitelman syndrome
Bartter syndrome
Familial magnesium malabsorption
Familial hypomagnesemia with hypercalciuria and nephrocalcinosis
Early-onset diabetes mellitus (caused by HNF1-beta mutation)
Autosomal recessive isolated hypomagnesemia (caused by epidermal growth factor mutation)
Autosomal dominant isolated hypomagnesemia (caused by Na-K-ATPase gamma subunit, Kv1.1, and cyclin M2 mutations)
Hypomagnesemia with secondary hypocalcemia (caused by TRPM6 mutation)
Endocrine causes
Hypercalcemia
Hyperparathyroidism
Hypoparathyroidism
Hyperthyroidism
Hyperaldosteronism
Diabetes mellitus
Syndrome of inappropriate antidiuretic hormone secretion
Hypomagnesemia secondary to decreased intake
Malnutrition
Starvation
Alcoholism, cirrhosis
Anorexia nervosa
Terminal cancer diseases
Critically ill patients receiving total parenteral nutrition
Pregnancy
Transdermal loss
Excessive sweating with inappropriate electrolyte substitution
Severe burns
Chelation due to extracorporeal circulation
Citrate anticoagulation
Cardiopulmonary bypass
Hypomagnesemia secondary to the following drugs
Thiazide diuretics, less so loop diuretics
Proton pump inhibitors
Digitalis
Vitamin D intoxication
Laxatives
Pamidronate
Antibodies: Cetuximab, matuzumab, and panitumumab
Immunosuppressants: Cyclosporin, tacrolimus, and ritodrine
Beta adrenergic agonists: Theophylline, salbutamol, and riniterol
Cytotoxic drugs: Cisplatin, carboplatin, and gallium nitrate
Antimicrobial agents
Aminoglycosides: Gentamicin, tobramycin, and amikacin
Antituberculosis drugs: Viomycin, and capreomycin
Amphotericin B
Pentamidine
Foscarnet

*Note:* Some elements may overlap or can be sorted into multiple groups.

The summary of inherited disorders affecting calcium and magnesium homeostasis was beyond the scope of this review. In detail, these are described elsewhere.^7^, ^8^, ^9^, ^10^

### Regulation of PTH Secretion by Calcium and Magnesium by the Parathyroid Gland in Physiologic Conditions

In its intact form, PTH consists of 84 amino acids with a molecular weight of 9.4 kDa.^11^ It increases the systemic Ca^2+^ concentration when binding to PTH receptors in many tissues of the body to mediate calcium homeostasis; the half-life of PTH is ∼4 minutes.^11^ For rapid modulation, PTH is stored in secretory vesicles of the parathyroid cells.

Acute forms of hypoparathyroidism are common in patients after parathyroidectomy that in severe cases may lead to acute hypocalcemia due to a phenomenon named the hungry bone syndrome caused by chronic bone calcium release, most likely due to primary or secondary hyperparathyroidism, with the latter usually being associated to advanced stages of chronic kidney disease (CKD).^12^

There is tight interaction of PTH and vitamin D for the regulation of magnesium uptake by the intestine and the kidney. Specifically, PTH stimulates renal 1,25-dihydroxy vitamin D_3_ (1,25[OH]_2_D_3_) production, which subsequently induces small intestinal Ca^2+^ and Mg^2+^ absorption and renal tubular reabsorption.^13^^,^^14^ Fibroblast growth factor 23 (FGF23) produced in the bone, and Klotho play an important role in the fine-tuning of 1-α-hydroxylase and thus in the sufficient but not excessive formation of 1,25(OH)_2_D_3_. As a negative feedback regulator, FGF23 downregulates 1,25(OH)_2_D_3_-induced intestinal Ca^2+^ absorption. Recent data indicate that FGF23 might also have a systemic regulatory role inhibiting intestinal Mg^2+^ absorption.^15^

Severe magnesium deficiency may be the cause of hypocalcemia in humans. The only known mechanism to modulate local and consecutive systemic fluctuations in Ca^2+^ and Mg^2+^-concentrations is the Ca^2+^-sensing receptor (CaSR), which belongs to the superfamily of guanine nucleotide-binding protein-coupled receptors (G-protein). It is mainly expressed by the principal cells of the parathyroid gland and renal tubule cells but is also present in other organs. Like all other G-protein-coupled receptors it consists of 7 circularly arranged transmembrane domains.

**Figure 1:**
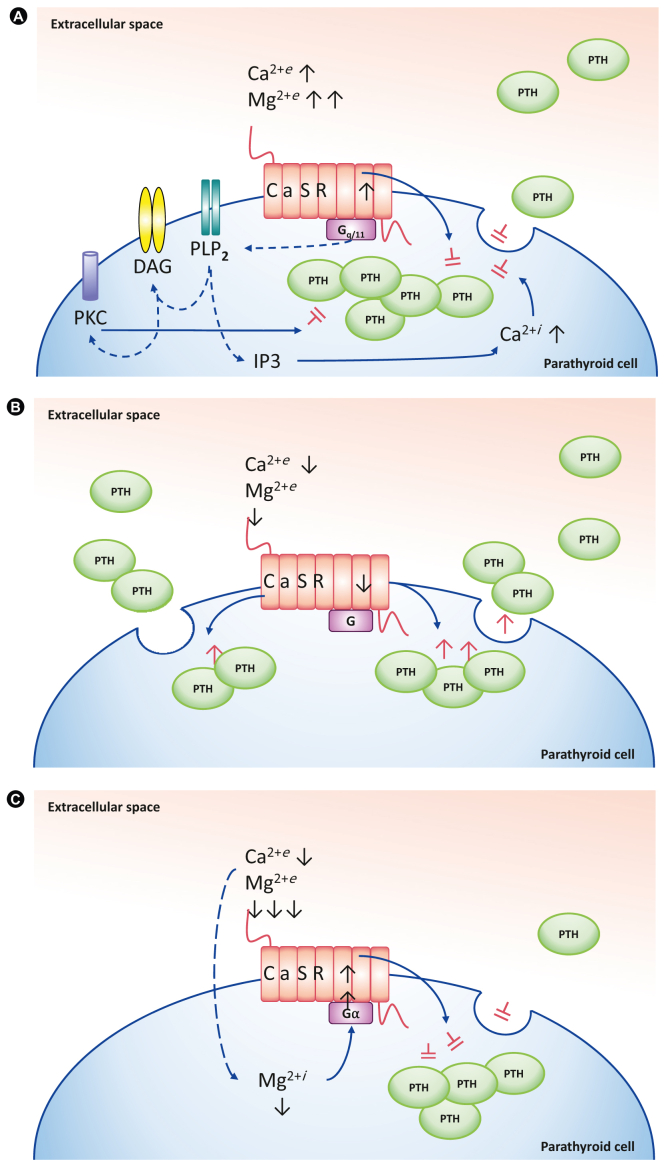
A, the inhibition of parathyroid hormone (PTH) release due to negative feedback is regulated by an increase of extracellular ionic calcium (Ca^2+^) or magnesium (Mg^2+^). In its active state, the calcium-sensing receptor (CaSR) couples with a heterotrimeric guanine nucleotide-binding protein (G-protein) activating the intracellular inositol-phosphate–pathway. This cascade includes diacylglycerol (DAG) and inositol-trisphosphate (IP3). This second messenger pathway modulates an increase in intracellular Ca^2+^ and together with proteinkinase–C (PKC) the PTH secretion is downregulated. B, disinhibition of the CaSR and consecutive modulation of PTH release regulated by a decrease of extracellular Ca^2+^ and Mg^2+^ appropriate to the prevailing extracellular calcium concentration. C, the hypomagnesemia paradox: Effective control of intracellular Mg regulation in parathyroid cells is still poorly understood. Attributed secondary to the low systemic Mg^2+^ concentration, intracellular hypomagnesemia develops that in return modulates disinhibition of the Gα subunit of the CaSR G-protein via a magnesium-binding site. Consequently, the CaSR is activated moderating the regular feedback mechanism leading to downregulation of PTH secretion to an inappropriately normal or low PTH level with respect to the systemic condition with hypocalcemia.^38^

In situations with sufficient calcium supplementation, increased binding of Ca^2+^ ions to the extracellular domain leads to a conformational change of the CaSR.^16^ An intracellular cascade by G-protein dependent stimulation, via Gq/11 activates intracellular phospholipase–C that subsequently cleaves phosphatidylinositol-4,5-bisphosphate into diacylglycerol and inositol-trisphosphate (IP3).^7^ Diacylglycerol activates proteinkinase–C downregulating the cellular hormone response, whereas IP3 leads to Ca^2+^-release at the endoplasmic reticulum, followed by an increase in the calcium concentration in the cytoplasm.^16^ This increase in intracellular Ca^2+^ concentration equally inhibits vesicle fusion and reduces the amount of exocytosis of PTH (Fig 1A).^7^ In hypercalcemic conditions, CaSR inhibits PTH release and stimulates the secretion of calcitonin by thyroid C-cells. In hypocalcemic conditions the inverse effect with reduced Ca^2+^ affinity on the CaSR leads to CaSR activation, consecutively modulating an increase in vesicular formation with PTH release from the parathyroid gland (Fig 1B).

### Case Summaries

The current reference interval for Mg^2+^ is suggested as 0.75 to 0.96 mmol/L with the mean being 0.85 mmol/L.^17^ In the literature Mg^2+^ has been attributed the forgotten electrolyte,^18^ which may reflect that it is not common knowledge that hypomagnesemia may induce a functional hypoparathyroidism.^4^ This condition is described by hypocalcemia in the presence of low or inappropriately normal PTH-levels secondary to hypomagnesemia. Interestingly symptomatic hypomagnesemia is usually not seen until serum magnesium decreases to ∼0.5 mmol/L or lower.^19^^,^^20^

For the first time in 1972, Anast et al^20^ reported on a patient with hypomagnesemia with paradoxical inadequacy of PTH levels in hypocalcemia-induced tetany. Jacob et al^21^ recently described a similar case affected by cardiac arrest of unknown cause when paradox hypoparathyroidism with severe hypomagnesemia, hypocalcemia and hypokalemia was detected and treated with intravenous magnesium, oral calcium, and vitamin D supplements.^21^ Others report on a case with a recently found mutation of the Transient receptor potential channel subfamily M, member 6 gene coding for a magnesium-selective ion channel that facilitates the transcellular reabsorption of magnesium from the urine,^9^^,^^22^ causing hypomagnesemia by excessive renal excretion of magnesium.^23^ A case series by Leicht et al^24^ summarizes on 4 patients with severe hypomagnesemia, hypocalcemia, and inadequate hypoparathyroidism in 3 patients with shortened bowel syndrome and one with alcoholism.^24^

### Effects of Impaired Kidney Function on Magnesium Hemostasis

Both experimental and clinical studies explored the relationship between serum magnesium levels, kidney function impairment, and renal outcomes in CKD.^25^

Magnesium deficiency may be observed at any stage of CKD^26^ and attention to secondary hyperparathyroidism must be paid. Magnesium is essential for vitamin D metabolism, specifically for 1α-hydroxylase, which activates vitamin D in the kidney. Low magnesium may therefore contribute to reduced activation of vitamin D, potentially worsening secondary hyperparathyroidism, preventing hypomagnesemia and hypocalcemia in CKD may therefore prevent the downregulation of key parathyroid receptor expression, such as calcium-sensing receptors, vitamin D receptor, and FGFR-Klotho receptor system, respectively.^27^

In CKD stage 3, down to an eGFR of ∼30 mL/min/1.73m^2^ one can typically compensate for declining kidney function by increasing the fractional excretion of magnesium, thus maintaining normal serum levels. Therefore, in general supplementation within the recommended dietary allowance by the European Food Safety Authority and United States Food and Nutrition Board, which is 300-320 mg and 350-420 mg/day in adult women and men, respectively, seems to be safe,^26^^,^^28^ with no significant risk of severe hypermagnesemia or adverse effects on bone metabolism.^29^ However, with progressive worsening of CKD in stage 4, compensatory mechanisms become insufficient, and patients with eGFR below 10 mL/min/1.73m^2^ are at high risk of developing hypermagnesemia.^30^ In patients undergoing hemodialysis and peritoneal dialysis, an inverse and independent relationship between serum magnesium and PTH levels, even after adjusting for calcium and phosphorus concentrations was seen previously.^31^^,^^32^ Studies examining the relationship between PTH and magnesium were summarized recently; however, most lack proper control or have methodological limitations, making it difficult to draw definitive conclusions regarding the optimal range for patients with CKD or dialysate concentrations for those requiring dialysis.^30^

### Mechanism of Peripheral PTH Resistance Associated to Hypomagnesemia

Parathyroid hormone resistance and impairments in the PTH signaling pathway have historically been categorized under the term pseudohypoparathyroidism. As outlined above, the parathyroid gland depends on magnesium for the synthesis and secretion of PTH. However, because the end-organ effects of PTH in the kidney and bone depend on magnesium as well, hypomagnesemia leads to hypocalcemia by causing both hypoparathyroidism and refractoriness of the target tissues to PTH (ie, PTH resistance), which cannot be resolved with calcium or vitamin D supplementation alone. The suggested mechanism is due to an impaired response of cyclic adenosine monophosphate to PTH.^33^^,^^34^

Allgrove et al^35^ report that in cases of moderate hypomagnesemia, where PTH secretion is only partially impaired, hypocalcemia may be further exacerbated because the moderately elevated PTH leads to a reduced responsiveness to its own target effects: Responsiveness to PTH was measured by the increase in plasma cyclic 3'5'-adenosine monophosphate after PTH injection. The increase in plasma cyclic adenosine monophosphate showed an inverse correlation with magnesium and PTH levels. This suggests that PTH concentration, rather than the severity of hypomagnesemia, plays a key role in determining the responsiveness of target tissues to PTH during magnesium deficiency.^35^ In addition, a reduction in extracellular magnesium levels was found to impair the skeletal response to PTH and 1,25(OH)_2_D_3_ in rat bone organ cultures in vitro.^36^ It was suggested that these effects in orchestration may play a role in hypocalcemia associated with magnesium depletion in vivo.^37^

### Mechanism Underlying the Inadequate Parathyroid Hormone Secretion in Hypomagnesemic Conditions

**Figure 2:**
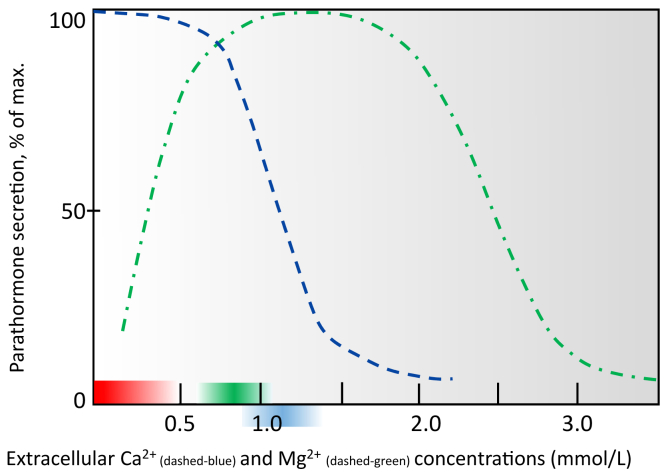
Relationship of extracellular Mg^2+^ and Ca^2+^ concentrations and parathyroid hormone (PTH) secretion of the parathyroid gland adapted from references.^4^^,^^7^ Reference interval for Mg^2+^ are 0.75 to 0.96 mmol/L (green) with the mean being 0.85 mmol/L; with the suggested range of risk for symptomatic hypomagnesemia < 0.5 mmol/L (red), Ca^2+^ reference interval 1.05 to 1.3 mmol/L (blue).^4^

Apparently, Ca^2+^ and Mg^2+^ in physiologic conditions are independently able to stimulate the CaSR. High concentrations of both, Ca^2+^ and Mg^2+^ lead to an inhibition of PTH (Fig 1A). Specifically, however, Mg^2+^ has divalent effects on the subsequent expectable PTH release, as very low Mg^2+^ concentrations will result in the opposite of the expected effect (Fig 2).

Only recently Quitterer et al^38^ discovered the underlying mechanism of this condition referred to as a paradoxical inhibition or inadequacy of PTH secretion. Despite previous suggestions on the pathogenesis, they found that the extracellular binding site of the CaSR was not involved in the hypoparathyroidism paradox, but rather intracellular depletion of Mg^2+^ interacted with the magnesium-sensitive intracellular part of the CaSR, specifically at the inhibitory Gα-subunit of the CaSR.^38^ Acknowledging that magnesium suppresses the guanine nucleotide exchange of small guanosine triphosphate-binding proteins,^39^ suggested the possibility of a similar mechanism for Gα subunits of the CaSR. In an in vitro model involving wild-type and mutant Gα, the latter with a decreased affinity for magnesium, they were able to demonstrate that the intracellular magnesium-binding site at the Gα subunit of the CaSR G-protein was responsible for disinhibiting the Gα-receptor, and the consecutive enhancement of the G-protein-mediated activation under low intracellular magnesium levels.^38^ Activation of the CaSR will then moderate the paradox feedback mechanism, leading to impaired PTH secretion as illustrated in Fig 1C.^40^

Unfortunately, the control mechanisms of intracellular Mg are still poorly understood, but very limited chemical Mg^2+^ gradient across the cell membrane and the membrane of cellular organelles is suggested.^41^ In contrast to other ions, such as in internal potassium balance,^42^ hypomagnesemia due to intracellular shift tends not to occur, underlining the severity of intracellular hypomagnesemia within the hypoparathyroidism paradox. Also, only limited information on cytosolic or intra-organelle protein’s ability to bind substantial amounts of Mg^2+^ under physiologic conditions is available.

Other than the aforementioned mechanism, the presence of Mg^2+^ binding sites has been reported for several other cellular proteins.^41^ As adjunct to the mechanism described above,^38^ in vitro experiments were performed by Rodríguez-Ortiz et al^27^ incubating intact rat parathyroid glands in different calcium and magnesium concentrations. They found that Mg^2+^ reduced PTH secretion mainly if serum Ca^2+^ was somewhat below normal. Specifically, the magnesium concentrations were inversely proportional to the release of PTH for low calcium concentrations, whereas for higher calcium concentrations their effect on PTH release was less evident.^27^ In addition, Mg^2+^ upregulated pivotal parathyroid CaSR, the vitamin D receptor, Klotho, and fibroblast growth factor receptor 1 at messenger-RNA and protein levels.^27^ Their results allow the following conclusion: As previously suggested by Brown et al^4^ Ca^2+^ is more potent than Mg^2+^ in suppressing PTH.^27^ This result acknowledges findings from Ferment et al^43^ performed in vivo. Of interest and in some contradiction to previous studies with normal to high Ca^2+^ concentrations by Browns’ group illustrated in Fig 2,^4^ only an extremely high Mg^2+^ concentration of 5.0 mmol/L was able to decrease PTH secretion in the rat model.^27^ Rat parathyroid glands were sensitive to an inhibitory effect of Mg^2+^ only when a moderate low calcium concentration was present.^27^

The authors discuss that avoiding hypomagnesemia and hypocalcemia in CKD may prevent downregulation of key parathyroid receptors, which could help to control for secondary hyperparathyroidism in progressive stages of CKD.^27^ Though magnesium is also required for the sensitivity of the target tissues to PTH,^27^ which emphasizes the multilocular complexity of the magnesium interaction.

## Discussion

Magnesium is a potentially overlooked but essential cation fulfilling pivotal roles in numerous cell functions, with symptoms appearing only when plasma levels are too low. The clinical signs and symptoms of hypomagnesemia may range from mild tremors to life-threatening cardiac arrythmias and neurologic complications with seizures.

Besides inherited disorders hypomagnesemia is most frequently caused by renal wasting, gut malabsorption, or decreased intake secondary to medication or redistribution from the extracellular to the intracellular compartment. A summary of causes underlying hypomagnesemia is presented in Table 1.

The paradox inadequate parathyroid secretion secondary to hypomagnesemia is a rare and not well understood phenomenon, and diagnosis and treatment requires special attention to differential diagnostics and treatment that we elaborate on in the following paragraphs.^44^

### Implications for Diagnostic Procedures

Magnesium homeostasis involves the ileum and the colon as the primary enteral sites. The kidney modulates Mg^2+^ reabsorption in the proximal tubule and the thick ascending loop of Henle, reabsorbing up to 60%-70% of the filtered magnesium, and the distal tubule.

Most frequent underlying conditions of hypomagnesemia are related to either reduced dietary intake, congenital or acquired defects modulating increased enteral or renal loss, diabetes, alcoholism, or drug-related interactions resulting in reduced absorption or increased excretion.

Magnesium excretion in the urine should therefore be assessed at initial presentation. In healthy individuals, magnesium excretion in 24-hour urine is 4-5 mmol. If excretion is less than 2 mmol/24h, there is a reasonable suspicion of a magnesium deficiency, whereas urinary Mg of >5 mmol/day are indicative of renal loss.^45^ The assessment of the urinary fractional magnesium excretion from spot urine is also an important tool to assess the renal etiology of hypomagnesemia and can be calculated using formula 1, where U and P correspond to the urinary and plasma concentrations of magnesium (Mg) and creatinine (Cr). Plasma magnesium in the denominator is multiplied by factor 0.7 to estimate the amount of plasma magnesium that is filtered, acknowledging that 30% of magnesium is protein bound.

$$\text{formula 1:}\;\text{FE : Mg}\;\left(\%\right)=\left[\left({\text{Mg}}_{U}\times C_{P}r\right)\;/\;\left(0.7\;\times {\text{Mg}}_{P}\times {\text{Cr}}_{U}\right)\right]\times 100$$

Bone is considered the primary storage site for both calcium and magnesium containing approximately 50%-65% of total body magnesium that is estimated around 1,000 mmol (∼25 g) in a 70 kg adult.^17^ About one-third of total magnesium resides in the muscles. Only about 0.3%-1% of magnesium is in extracellular circulation and therefore magnesium values within the normal serum range do not rule out the possibility of total body deficiency compensated for by the release of Mg^2+^ from the bone pool.^17^ The estimation of total magnesium status remains a challenge, as a significant amount is stored as surface substituents of the hydroxyapatite mineral component of the bone, and thus magnesium is not completely bioavailable during magnesium deprivation.^46^

In the case of severe or refractory hypocalcemia, we recommend the complementary measurement of serum albumin, ionized-bound calcium, and albumin-bound calcium, magnesium, PTH, and calcitonin and the assessment of vitamin D status, kidney function, and blood glucose status, including hemoglobin A1c. Blood-gas analysis should be performed to assess the patients’ acid-base status. Additional urinalysis should include point of care dipstick-analysis, complemented by measurement of urinary electrolytes, specifically urinary Mg and proteinuria.

Hypokalemia may frequently coincide with hypomagnesemia, and we therefore suggest assessment of a complete routine serum electrolyte profile.

### Implications for Therapy

The treatment of hypomagnesemia should be based on the severity of symptoms and the severity of magnesium depletion. Clinically applicable grades for classification of the severity of hypomagnesemia are available from the US National Cancer Institute, summarized in Table 2.^47^

**Table 2:** Grades of Severity of Hypomagnesemia According to U.S. Department of Health and Human Services^47^

Grade	Magnesium Serum Concentrations in mg/dL	Magnesium Concentrations in mmol/L	Severity
Grade 1	< LLN-1.2 mg/dL	< LLN-0.5 mmol/L	Mild
Grade 2	< 1.2-0.9 mg/dL	< 0.5-0.4 mmol/L	Moderate
Grade 3	< 0.9-0.7 mg/dL	< 0.4-0.3 mmol/L	Severe
Grade 4	< 0.7 mg/dL	< 0.3 mmol/L	Life-threatening
Grade 5	Death related to or due to associated adverse event		

Abbreviations: LLN, lower limit of normal.

Of clinical interest, hypocalcemia in conditions with severe magnesium deficiency cannot be treated or corrected with supplementation of calcium, vitamin D, or both.^18^ Repeated intravenous (i.v.) injection of magnesium sulfate 50% (equivalent to 486 mg/20 mmol of magnesium) may be necessary to achieve a stable increase of Ca^2+^, and consecutive normalization of PTH levels. In analogy to the hungry-bone phenomenon observed with calcium, we suggest that available magnesium bone reserves may be severely depleted in patients with persistent magnesium loss that, when substituted, may lead to a rapid and sustained Mg^2+^ absorption to replenish bone reserves. Severe serum albumin reduction may be of clinical relevance, and supplementation may be considered to increase serum magnesium-binding and calcium-binding capacity. Still, clinician awareness of kidney function in the setting of electrolyte imbalance is necessary, as they may be at risk of hypermagnesemia when supplementation is performed uncontrolled because renal excretion of potentially excessive magnesium supplementation may be limited in patients with CKD.

If chronic supplementation is needed due to renal magnesium wasting, patients may benefit from amiloride, which is a potassium-sparing and magnesium-sparing diuretic; however, the magnesium-sparing effect may be limited with studies suggesting an increase of 0%-5% in serum Mg^2+^ levels.^48^ Use of thiazide diuretics should be avoided because their use is found to be associated with lower serum magnesium levels and an increased risk of hypomagnesemia development.^49^ Recent findings by Nijenhuis et al^50^ indicate that enhanced passive Ca^2+^ transport in the proximal tubule due to extracellular volume contraction may be responsible for the hypocalciuria that develops during chronic thiazide treatment, while specifically reduced renal expression levels of the epithelial transient receptor potential Mg^2+^(–ion) channel (TRPM6) were found in their mouse models apparently responsible for inappropriately high fractional Mg^2+^ excretion into the urine.

**Figure 3:**
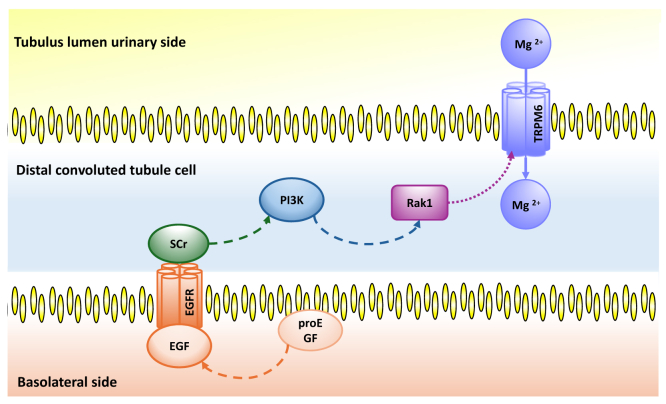
The transient receptor potential channel subfamily M, member 6 (TRPM-6) gene encodes for a magnesium-selective ion channel that facilitates the transcellular reabsorption of magnesium from the urine. In the nephron TRPM6 is found in the apical membrane of the distal convoluted tubule, only and is responsible for ∼5%-15% of total magnesium reabsorption, whereas in the intestine TRPM6-expression is highest in the colon and caecum.^57^ The TRPM6 channel consists of 6 membrane-spanning domains forming a pore-region modulating Mg^2+^ reabsorption by means of a potassium-channel induced negative membrane potential.^58^ It is suggested, that TRPM6 requires TRPM7 to function; however, at present, the latter is not well understood. Transcription of TRPM6 is regulated by epidermal growth factor (EGF) as a magnesiotropic hormone, thus mutations to the EGF-gene themselves also cause hypomagnesemia.^59^ Pro-EGF is expressed both on luminal and basolateral membranes of distal convoluted tubule cells and releases the active form of the EGF hormone^10^ interacting with the EGF-receptor on the basolateral side in turn activating an Src-family kinase and PI3K/Rac1 pathway to increase expression of TRMP6.^56^

Patients with diabetes mellitus also frequently develop hypomagnesemia, which has been attributed to diabetic hyperfiltration and the interaction of insulin in downregulating TRPM6 activity and expression.^51^ Recent studies point toward a beneficial double action of sodium glucose linked transporter 2-inhibitors for the treatment of hypomagnesemia, specifically targeting the mechanism of urinary magnesium wasting.^52^ Targeting peripheral insulin resistance in diabetes mellitus may potentially point toward improved serum Mg^2+^ levels by increased expression of the TRPM6,^51^ however, improvements in magnesium levels have also been reported in patients without diabetes.^53^ In addition, a magnesium shift from the intracellular to the extracellular compartment is suggested by a reduction of circulating insulin levels due to their glucosuric effects.^54^ Others report on an increase of epidermal growth factor mRNA in distal convoluted tubular epithelial cells that is known to increase TRPM6 expression acting as a magnesiotropic hormone (Fig 3).^55^, ^56^, ^57^, ^58^, ^59^ Of interest, the overall magnesium-sparing effect seems to be more pronounced in patients with hypomagnesemia refractory to traditional therapy, suggesting beneficial use of sodium glucose linked transporter 2-inhibitors and potential for future research.

The initial magnesium supplementation in hypomagnesemia in patients at risk of adverse events is summarized in Table 3 as suggested by Parikh et al^60^ Ad hoc supplementation of 1-2 g magnesium sulfate (4-8 mmol) i.v. over 2-15 minutes are suggested in hemodynamically unstable patients.^44^ Subsequent higher doses of 20 mmol up to 40 mmol mixed in 100 mL to 1 L sodium chloride 0.9% or glucose 5% solution may be given over 2-4 hours using a central vein catheter under appropriate monitoring when exceeding rates of 8 mmol/hour. The maximum rates should not exceed 36 mmol/hour. When the patient has coinciding hypokalemia in general, magnesium should be substituted first. Patients with moderate hypomagnesemia of < 0.5 mmol/L usually receive an initial substitution of 20 mmol at our center with adaptation to individual needs after subsequent controls.

**Table 3:** Treatment of Hypomagnesemia in Patients at Risk of Adverse Events

Urgency	Suggested Magnesium Supplementation
Emergency	10-20 mmol i.v. followed by 40 mmol over 4 hours i.v.
Critically ill	40 mmol i.v. on day 1, and 10-20 mmol on days 2-5 i.v.
Less severely ill	15 mmol per day

Abbreviation: i.v., intravenous.

Additional supplementation of cholecalciferol, in our experience led to an increase in 25-OH vitamin D levels but not to an increase in serum-Ca^2+^, which is expected, because magnesium deficiency inherently decreases responsiveness to vitamin D.^61^ On the contrary, renal resistance to PTH would not result in serum vitamin D increase.^5^ In contrast Leicht et al^24^ report, that in 4 cases with PTH being immeasurably or inadequately low, magnesium supplementation returned PTH to normal or adequately elevated values, with serum calcium rising to normal levels within 2-5 days.^24^

They also noted that vitamin D levels did not normalize, highlighting the need for additional cholecalciferol supplementation in cases of low 25-OH vitamin D levels. Similarly, Rude et al^62^ observed that while magnesium replacement corrected plasma calcium and PTH levels, it did not restore calcitriol concentrations, the underling effects however are unclear.

### Interdisciplinary Treatment

Thorough interdisciplinary diagnostic and interprofessional care management should be sought, including clinicians and pharmacists. Monitoring of laboratory parameters are frequently directed to nurses.^63^ The consultation of a nephrologist should be considered specifically when inherited tubular disorders are suspected or when magnesium metabolism is disproportionately difficult to assess or to regulate with appropriate supplementation. Cardiologists should be consulted in the presence of cardiac arrhythmias from magnesium and concurrent calcium deficiency, and patients monitored using telemetry. Gastroenterologists will assist in the diagnostic of enteral malabsorption and perform endoscopy procedures. Consultation with a pharmacist is recommended in patients with polypharmacy or uncertainty of interactions to assist in determining medication associated with hypomagnesemia.

### Patient Prognosis

The prognosis of hypomagnesemia depends on the underlying clinical condition, and the clinician’s experience in the differential diagnostic and therapy of electrolyte disorders and those associated with dysfunction of the parathyroid hormone axis. As illustrated, severe hypomagnesemia may be a life-threatening condition, especially if treatment options are not recognized and implemented in a timely manner. Patients presenting with critical illness frequently associated with arrythmias, neurologic manifestations or the need for mechanical ventilation may have increased risk of adverse outcome.^64^

Patients in whom an underlying pathology was identifiable will have good prognosis, especially if a reversible condition was present. Patients with inherited disorders may benefit from regular visits to an appropriate specialist for endocrine and hereditary diseases. In some cases, regular supplementation of magnesium and regular control of magnesium levels and the parathyroid-mineral-bone axis may be necessary. Further regular treatment by a nephrologist or osteologist may therefore be advisable.

## Conclusion

Because of the rarity and complexity of the interaction of magnesium with the PTH secretion and high interdisciplinary clinical relevance, this review aims to raise awareness to the phenomenon of paradoxical hypomagnesemia in cases with hypocalcemia. This review found that primary studies on the regulatory effects of magnesium on PTH secretion published in the literature are limited. Effective control of intracellular magnesium regulation in parathyroid cells is still poorly understood. Future research should therefore address the magnitude and relevance of intracellular hypomagnesemia on parathyroid and other cells, such as cardiomyocytes, in severe forms of systemic magnesium deficiency.

In case of severe hypomagnesemia, extended therapeutic intervention should include primary substitution of magnesium next to potassium, calcium, and vitamin D supplementation, according to the laboratory findings.
